# Dietary diversity and meal frequency among infant and young children: a community based study

**DOI:** 10.1186/s13052-017-0384-6

**Published:** 2017-08-15

**Authors:** Aysheshim Kassahun Belew, Bekrie Mohammed Ali, Zegeye Abebe, Berihun Assefa Dachew

**Affiliations:** 1 Department of Human Nutrition, Institute of Public Health, College of Medicine and Health Sciences, University of Gondar, Gondar, Ethiopia; 2 Department of Epidemiology and Biostatistics, Institute of Public Health, College of Medicine and Health Sciences, University of Gondar, Gondar, Ethiopia

**Keywords:** Children, Dietary diversity, Meal frequency, Ethiopia

## Abstract

**Background:**

Insufficient quantities, frequencies, and inadequate quality of complementary feedings have a negative effect on child health and growth, especially in the first two years of life. Therefore, the aim of this study was to assess the minimum dietary diversity, meal frequency and its associated factors among infants and young children aged 6–23 months at Dabat District, northwest, Ethiopia.

**Methods:**

A community- based cross-sectional study was conducted from February 15 to March 10, 2016. The simple random sampling method was used to select study participants. An interviewer- administered structured questionnaire was used to collect data. Both Crude and Adjusted Odds Ratio with the corresponding 95% confidence interval were calculated to show the strength of association. In the multivariable analysis, variables with less than 0.05 *P*-value were considered statistically significant.

**Results:**

The proportion of children who met the minimum dietary diversity and meal frequency were 17% (95% CI: 14.9, 19.4%) and 72.2% (95% CL: 69.3, 75%), respectively. Satisfactory media exposure (AOR = 2.79; 95% CI: 1.74, 4.47), postnatal care visits (AOR = 1.96; 95% CI: 1.32, 2.88), participation in child growth and monitoring follow ups (AOR = 1.65; 95% CI: 1.14, 2.39), age of children (AOR = 2.34; 95% CI: 1.33, 4.11) and age of mothers (AOR = 1.89; 95% CI: 1.09, 3.27) were positively associated with dietary diversity. Similarly, age of children (AOR = 2.38; 95% CI: 1.56, 3.65), household wealth status (AOR = 1.84; 95% CI: 1.27, 2.68), residence (AOR = 3.02; 95% CI: 1.41, 6.48), sources of information (AOR = 1.72; 95% CI:1.14, 2.59) and participation in child growth monitoring folow ups (AOR = 1.57; 95% CI: 1.13, 2.19) were significantly associated with meal frequency.

**Conclusion:**

In this study, the proportion of children who received the minimum dietary diversity and meal frequency were low. Media exposure, age of children, postnatal care visits, and participation in child growth and monitoring follow-ups were significantly associated with dietary diversity. Likewise, wealth status and residence had a significant association with meal frequency. Thus, encouraging all mothers to participate in child monthly growth monitoring programs, intensive media advertising and strengthening counseling of mothers, and postnatal care visit are highly recommended for achieving the recommended dietary practices.

## Background

Complementary feeding is the process of introducing other liquid and solid foods in addition to breastmilk when the energy demand of growing children does not maintain by breastmilk alone [1]. As infants and young children are found in dynamic growth and development, the World Health Organization (WHO) has recommended that solid, semisolid or soft foods should introduced at the age of six months [2–4], and that infants and young children should get the minimum meal frequency, dietary diversity, acceptable diet, and iron rich foods [5].

Globally, only 60% of infants start complementary feeding between the ages of 6 to 9 months. This indicates that millions of infants are given complementary foods either too early or too late [6]. For instance, in Africa less than one-third and around one-half (50%) of the 6 to 23-months-old children meet the minimum criteria for dietary diversity and meal frequency, respectively [7]. In addition, only less than 20% of the children receive adequate complementary feeding. That is why the level of stunting in most African countries increases threefold during the two years of life [8].

Appropriate child feeding practice is vital in the first two years of life because malnutrition is common, especially in this age group. In addition, children become vulnerable to growth retardation, delayed mental development, micronutrient deficiencies, and common childhood illnesses and dealth [9, 10]. Moreover, inappropriate complementary feeding practices increase under five mortality by six-folds [11].

In Ethiopia, inappropriate complementary feeding practices were account for 57% of the deaths of under-five children, and 8.3% current workforce loss [12]. It has also contributed to 40% child stunting, 9% child wasting, and 25% child underweight in Ethiopia [13].

Different studies have documented that dietary diversity and meal frequency are associated with socio-demographic characteristics. Accordingly, low levels of parental education, and age of mothers [14–16], maternal occupation [17], age and birth order of children [14, 15], household wealth status [14, 16], family size [14], and residence [15] were significantly associated with minimum dietary diversity practices . Furthermore, birth interval [18], satisfactory media exposure [14, 15], mother’s involvement in decision making [15], maternal antenatal (ANC) and postnatal (PNC) care follow ups [18, 19] were significantly associated with practicing minimum meal frequency.

Nowadays, the Government of Ethiopia is giving especial attention to child health and nutrition. For instance, it has endorsed the National nutrition program, introduced free of charge antenatal care services, assigned two health extension workers to each ʽKebeleʼ (The smallest administrative unit in Ethiopia), is used manual for complementary feeding practices, launched the monthly infant and child growth monitoring program and a continous media promotion on the importance of providing diversified and frequent diet to children. [20]. But according to the Ethiopian Mini Demography and Health Survey (EDHS) 2011 report, only 4.3% and 47.7% of children received the minimum dietary diversity and meal frequency, respectively [21].

As a matter of fact, is known about the dietary diversity and meal frequency practices in the northwestern part of Ethiopia, especially Dabat District. But, comprehensive data is needed for policy makers, governmental and nongovernmental organizations who are working on infant and young child health and nutrition. Therefore, it is important to investigate the practices of dietary diversity, meal frequency, and its associated factors among infant and young children.

## Methods

### Study setting and design

A community-based cross-sectional study was conducted from February 15 to March 10, 2016, at Dabat District, northwest Ethiopia. The district is located at 814 km away from Addis Ababa, the capital city of Ethiopia, and 75 km away from Zonal town, Gondar. The district has 6 health centers and 35 health posts. A total of 8875 under two years of children were living in the study area. The district has four urban and twenty-six rural kebeles.

### Sample size and sampling producer

All infants and young children 6–23 months old living in Dabat District were eligible for the study. The sample size was calculated using the single proportion formula by considering the following assumptions; the proportion of 12.6% for minimum dietary diversity [15], a 95% confidence level, and a 3% margin of error for dietary diversity. Finally, 1034 was adopted by considering, a 10% non-response rate and a design effect of 2. A multistage stratified sampling followed by the simple random sampling technique was employed to select the study participants. Initially, kebeles were stratified into urban and rural. Of the total 30 kebeles, nine (one urban and eight rural) were selected by the lottery method. Lists and the total number of infants and young children in all kebeles of the district were obtained from health extension workers. Then, the total number of infants and young children included in the study were proportionally allocated. Finally, the simple random sampling technique was used to select participants.

### Data collection tool and procedure

A structured interviewer-administered questionnaire was used to collect data. The questionnaire was adopted from EDHS 2011, WHO standardized questionnaire for IYCF practices, and other similar studies with some modification to fit the local context. The questionnaire was prepared in English and translated to Amharic and finally translated back to English to maintain consistency. A pretest was done on 5 % of the sample out of the study area. Two days training was given to data collectors and supervisors. A total of nine clinical nurse data collectors and three public health expert supervisors were recruited for the study. During the data collection period, a close supervision was done by the principal investigator and the supervisors, by overseeing how data collectors run the questions to the respondents and checking the collected data for completeness. Appropriate feedback was given before the next data collection period.

### Assessment of dietary diversity and meal frequency

Determination of the dietary diversity score (DDS) of each child was started by asking the mother to list all food consumed by the child in the 24 h preceding the survey. Then, the reported food items were classified into seven food groupsas grains, roots and tubers, legumes and nuts, dairy products (milk, yogurt, cheese), flesh foods (meat, fish, poultry and liver/organ meats), eggs, vitamin-A-rich fruits and vegetables,other fruits and vegetables. Children getting four or more food groups were classified as meeting the minimum dietary diversity; otherwise, they were considered as getting low minimum dietary diversity [2].

Similarly, meal frequency of the child was determined by asking the mother how many times the child took solid, semi-solid, or soft foods in the 24 h preceding the survey. Accordingly, two or more times for breastfed infants 6 to 8.9 months of age, three or more times for breastfed children 9 to 23.9 months, and four times for non-breastfed children 6 to 23.9 months were considered as the children received the minimum meal frequency [2].

### Assessment of media exposure

A woman aged 15 to 49 years and read a newspaper or magazine at least once a week or listened to the radioor watched television was considered as having a satisfactory media exposure.

Household wealth index was determined using Principal Component Analysis (PCA) by considering household assets, such as quantity of cereal products, house, livestock and agricultural land ownership. First, variables were coded between 0 and 1. Then, they were entered and analyzed using PCA, and variables with a communality value of greater than 0.5 were used to produce factor scores. Finally, the factor scores were summed and ranked into tertiles as poor, medium, and rich.

### Data processing and analysis

All of the returned copies of the questionnaire were manually checked for completeness and consistency of responses. Then, the collected data were entered into EPI-INFO version 7 and exported to SPSS version 20 for further analysis. The dietary diversity and meal frequency indicator was dichotomous variable classified into 0 and 1, representing those who met and those who did not meet the minimum dietary and the minimum feeding practices, respectively. Descriptive statistics were summarized by using figures, tables, and texts. Both the bivariable and multivariable logistic regression analyses were used to identify variables associated with dietary diversity and meal frequency. Variables with less than 0.2 *p*-values in the bivariable analyses were fitted into the multivariable logistic regression analysis. Both Crude Odds Ratio (COR) and Adjusted Odds Ratio (AOR) with the corresponding 95% Confidence Interval were calculated to show the strength of the association. Finally, in the multivariable analysis, variables with less than 0.05 *P*-values were considered as statistically significant.

## Results

### Socio-demographic characteristics

A total of 1034 infants and young children aged 6 to 23 months were enrolled along with their mothers. The mean age (±SD) of the mothers was 29.06 ± 6.58 years. Almost all, 1033 (99.9%), of the participants were Amhara by ethnicity and 996 (96.3%) were Orthodox chiristians. Two-thirds, 685 (68.2%), of the mothers were unable to read and write. About 947 (91.6%) of the mothers were housewives (Table 1).

**Table 1:** Parental level characteristics of children aged 6–23 months, Dabat, Northwest Ethiopia, 2016 (*n* = 1034)

Variables	Frequency	Percentage
Mother’s age		
15–24	22	2.1
25–34	525	50.8
35–50	487	47.1
Marital status		
Married	979	94.7
Separate	19	1.8
Divorced	17	1.6
Single	16	1.5
Widowed	3	0.3
Mother’s education		
Unable to read and write	685	68.2
Primary education	255	24.7
Secondary education	55	5.3
Higher education	39	3.8
Father’s education		
Unable to read and write	503	48.8
Primary education	413	40
Secondary education	71	6.9
Higher education	44	4.3
Mother’s occupation		
Housewife	947	91.6
Government employee	53	5.1
Merchant	22	2.1
Student	7	0.7
Daily laborer	5	0.5
Father’s occupation		
Farmer	814	79
Government employee	73	7.1
Daily laborer	66	6.4
Merchant	52	5.0
Self employee	26	2.5

### Child, household, and community-related characteristics

Of the total children, 527 (51%) were male. The mean (±SD) age of the children was 13.74 ± 5.31 months. Most, 1031 (97%), of the children were breastfed at the time of data collection. The majority of the mothers, 971 (93.9%), were involved in household decision making, and one-quarter, 269 (26.1%), were found in the poor wealth status (Table 2).

**Table 2:** Child, household and community characteristics of children aged 6–23 months, Dabat, Northwest Ethiopia, 2016 (*n* = 1034)

variables	Frequency	Percentage
Age of a child		
6–11 month	418	40.4
12–17 month	309	29.9
18–23 month	307	29.7
Birth order		
First	215	20.8
Second to fifth	675	65.3
Above fifth	144	13.9
Age when complementary feeding was started		
0–5 months	92	8.9
6 months	433	41.9
7–11 months	395	38.2
> =12 months	114	11
Number of under five children		
One	585	56.6
Two	442	42.7
Above three	7	0.7
Decision making at the household		
Mothers participated	971	93.9
Mothers not participated	63	6.1
Household wealth status		
Poor	269	26.1
Middle	415	40.1
Rich	350	33.8
Exposure to media		
Unsatisfactory	827	80
Satisfactory	207	20
Residence		
Rural	900	87
Urban	134	13
Home gardening		
No	838	81.04
Yes	196	18.96
Purpose of home gardening(*n* = 196)		
For household consumption	102	52.0
Both selling and for household consumption	90	45.9
For selling	4	2.1

### Child, and health care level related characteristics

More than half, (57.8%), of children were born at a health institution. Most, 872 (84.3%), of the mothers’ sources of information about IYCF practices were health professionals. This study also showed that two-thirds, 699 (67.6%), of the mothers attended four and above ANC follow-ups and 393 (38%) of the mothers had PNC visits**.**

### Dietary diversity and meal frequency practices

**Fig. 1:**
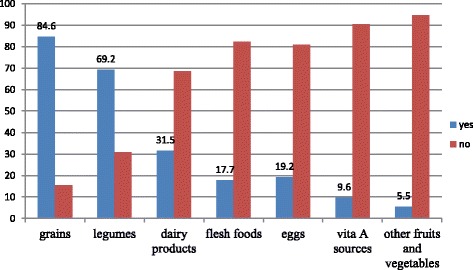
Types of food groups practiced among 6–23 months children, in Dabat district, Northwest Ethiopia, 2016

The proportion of children who received the recommended minimum dietary diversity and meal frequency was 17% (95% CI: 14.9, 19.4%) and 72.2% (95% CI: 69.3, 75%), respectively. Grains, tubers, and roots were provided to the greatest number of children (84.6%), and only a few (5.5%), were given other fruits and vegetables (Fig. 1).

### Factors associated with dietary diversity and meal frequency

The multivariate logistic regression output (Table 3) showed that postnatal care visits, participation in child growth monitoring follow ups, media exposure, age of mothers and children were significantly associated with minimum dietary diversity. Whereas, residence, sources of information, family wealth status and age of children were significantly associated with minimum meal frequency (Table 4).

**Table 3:** Factors associated with minimum dietary diversity practices among 6–23 months of children, Dabat district, Northwest Ethiopia, 2016

Variables	Minimum dietary diversity		Crude Odds Ratio with 95% CI	Ajdusted Odds Ratio with 95% CI
	Adequate	Inadequate		
	N (%)	N (%)		
Mother’s education				
Unable to read and write	92 (13.4)	593 (86.6)	1.00	1.00
Primary education	50 (19.6)	205 (80.4)	1.57 (1.08, 2.30)	1.24 (0.79, 1.95)
Secondary education	16 (29)	39 (71)	2.64 (1.40, 4.93)	1.30 (0.60, 0.80)
Higher education	18 (46.2)	21 (53.8)	5.53 (2.84, 10.76)	1.59 (0.66, 3.80)
Age of children				
6–11	17 (8.1)	192 (91.9)	1.00	1.00
12–17	33 (15.8)	176 (84.2)	2.12 (1.14, 3.94)	1.41 (0.73, 1.71)
18–23	126 (20.5)	490 (79.50)	2.90 (1.70, 4.95)	2.34 (1.33, 4.11)*
Birth order				
First	47 (21.9)	168 (78.1)	1.00	1.00
Second to fourth	95 (17.3)	453 (82.7)	0.75 (0.51, 1.11)	0.70 (0.41, 1.19)
Above fourth	34 (21.9)	237 (87.5)	0.51 (0.32, 0.83)	0.74 (0.37, 1.48)
Media exposure				
Unsatisfactory	101 (12.2)	726 (87.8)	1.00	1.00
Satisfactory	75 (36.2)	132 (63.8)	4.08 (2.87, 5.81)	2.79 (1.74, 4.47)*
Household wealth status				
Poor	32 (11.9)	237 (88.1)	1.00	1.00
Middle	65 (15.7)	350 (84.3)	1.37 (0.87, 2.17)	1.53 (0.94, 2.47)
Rich	79 (22.6)	271 (77.4)	2.16 (1.38, 3.37)	1.35 (0.81, 2.25)
Residence				
Rural	130 (14.4)	770 (85.6	1.00	1.00
Urban	46 (34.3)	88 (65.7)	3.10 (2.07, 4.63)	1.14 (0.62, 1.20)
Postnatal visit				
No	75 (11.7)	566 (88.3))	1.00	1.00
Yes	101 (25.7)	292 (74.3)	2.61 (1.88, 3.63)	1.95 (1.34, 2.88)*
Home gardening				
No	163 (16.5)	825 (83.5)	1.00	1.00
Yes	13 (28.3)	33 (71.7)	1.99 (1.03, 3.87)	1.88 (0.91, 3.92)
Place of birth				
Home	114 (64.8)	62 (35.2)	1.00	1.00
Health institution	484 (56.4)	374 (43.6)	0.70 (0.50, 0.98)	0.93 (0.63, 1.37)
participation in growth monitoring follow up				
No	78 (13.7)	493 (86.3)	1.00	1.00
Yes	98 (21.2)	365 (78.8)	1.70 (1.22,2.35)	1.65 (1.14, 2.39)*
Currently breastfed				
No	10 (32.3)	21 (67.7)	2.40 (1.11,5.19)	1.02 (0.24, 4.38)
Yes	166 (16.6)	837 (83.4)	1.00	1.00
Age of mothers				
15–24	36 (14.6)	210 (85.4)	1.00	1.00
25–34	107 (20.4)	418 (79.6)	1.49 (0.99, 1.39)	1.89 (1.09, 3.27)*
35–50	33 (12.5)	230 (87.5)	0.84 (0.50, 1.39)	1.07 (0.57, 2.14)

*indicate significant at *p* value less than 0.05 in multivariable logistic analysis

**Table 4:** Factors associated with minimum meal frequency practices among 6–23 months of children, Dabat district, Northwest Ethiopia, 2016

	Minimum meal frequency			
Variable	Yes N (%)	No N (%)	Crude Odds Ratio with 95% CI	Ajdusted Odds Ratio with 95% CI
Mother’s education				
Unable to read and write	475 (69.3)	210 (30.7)	1.00	1.00
Primary education	193 (75.7)	62 (24.3)	1.38 (0.99, 1.912)	1.39 (0.93, 2.10)
Secondary education	42 (76.4)	13 (23.6)	1.43 (0.75, 2.72)	1.01 (0.43, 2.38)
Higher education	37 (94.9)	2 (5.1)	8.18 (1.99, 34.25)	2.22 (0.37, 13.14)
Age of the child (months)				
6–11	86 (41.9)	123 (58.9)	1.00	1.00
12–17	139 (66.5)	70 (33.5)	2.84 (1.91, 4.228)	2.38 (1.56, 3.65)*
18–23	522 (84.7)	94 (15.3)	7.94 (5.58, 11.29)	8.03 (5.50, 11.73)*
Media exposure				
unsatisfactory	571 (69)	256 (31)	1.00	1.00
Satisfactory	176 (85)	31 (15)	2.55 (1.69, 3.83)	1.31 (0.77, 2.21)
Household wealth status				
Poor	169 (62.8)	100 (37.2)	1.00	1.00
Middle	296 (71.3)	119 (28.7)	1.47 (1.06, 2.04)	1.84 (1.27, 2. 68)*
Rich	282 (80.6)	68 (19.4)	2.45 (1.71, 3.53)	2.39 (1.57, 3.67)*
Residence				
Rural	625 (69.4)	275 (30.6)	1.00	1.00
Urban	122 (91)	12 (9)	4.47 (2.43, 8.23)	3.02 (1.41, 6.48)*
Postnatal visit				
No	426 (64.5)	215 (33.5)	1.00	1.00
Yes	321 (81.7)	72 (18.3)	2.25 (1.66, 3.05)	1.81 (1.26, 2.60)*
Receiving information on IYCF				
No	96 (59.3)	66 (40.7)	1.00	1.00
Yes	651 (74.7)	221 (25.3)	2.025 (1.43, 2.87)	1.72 (1.14, 2.59)*
Participation in growth monitoring follow ups				
No	379 (66.4)	192 (33.6)	1.00	1.00
Yes	368 (79.5)	95 (20.5)	1.96 (1.48, 2.61)	1.57 (1.12, 2.19)*
Husband’s education				
Unable to read and write	350 (69.7)	152 (30.3)	1.00	1.00
Primary education	298 (72)	116 (28)	1.11 (0.83, 1.47)	0. 88 (0.63, 1.23)
secondary education	55 (77.5)	16 (22.5)	1.48 (0.82, 2.67)	1.33 (0.58, 3.04)
College diploma &above	41 (93.2)	3 (6.8)	5.94 (1.81, 19.46)	3.27 (0.37, 28.75)
Father’s Occupation				
farmer	573 (67)	241 (33)	1.06 (0.46, 2.42)	1.83 (0.63, 5.31)
Government employee	66 (90.4)	7 (9.6)	4.19 (1.34, 13.11)	1.24 (0.31, 4.92)
Daily laborer	49 (74.2)	17 (25.8)	1.28 (0.47, 3.48)	1.89 (0.57, 6.31)
merchant	41 (78.8)	11 (21.2)	1.66 (0.57, 4.81)	1.15 (0.37, 3.52)
Self employee	18 (69.2)	8 (30.8)	1.00	1.00

*indicate significant at *p* value less than 0.05 in multivariable logistic analysis

Accordingly, mothers participating in growth monitoring follow-ups were 1.65 times (AOR = 1.65; 95% CI: 1.14, 2.39) more likely to provide minimum dietary diversity compared to their counterparts. Similarly, higher odds of receiving adequate dietary diversity were observed among mothers who had postnatal care visits (AOR = 1.95; 95% CI: 1.34, 2.88) and satisfactory media exposure (AOR = 2.79; 95% CI: 1.74, 4.47).

Likewise, mothers who lived in the urban area were about 3 times more likely to provide the recommended minimum meal frequency compared to mothers who lived in the rural area (AOR = 3.02; 95% CI: 1.41, 6.48). Mothers who obtained information from health professionals were 1.7 times more likely to provide minimum meal frequency compared to their counterparts (AOR = 1.72; 95% CI: 1.14, 2.59). Moreover, children belonging to middle and rich wealth status families were 1.8 and 2.3 times (AOR = 1.84; 95% CI: 1.27, 2.68) and (AOR = 2.40; 95% CI: 1.568, 3.69) more likely to receive the recommended minimum meal frequency, respectively, compared to poor wealth status families.

Finally, children in the 12–17 and 18–23 month age groups were 2.4 (AOR = 2.38; 95% CI: 1.56, 3.65) and 8 times (AOR = 8.03; 95% CI: 5.50, 11.73**)** more likely to receive the recommended number of feeds, respectively,  compared to children aged between 6 and 11 months.

## Discussion

This community-based cross-sectional study identified that the proportion of children who received the minimum dietary diversity was 17% (95% CI: 14.9, 19.4%). The finding is in line with the findings from Kenya (17.9%) [22] and Ethiopia (17.8%) [23]. But it is higher than the study reported in Zambia (12%) [24]. The difference might on the result of the designs used. The five year longitudinal design in Zambia, for example, had the capacity of showing actual and summarized feeding practices of children. The current finding is also higher than the 2011EDHS report of 10.8% [14]. This difference might be due to the fact that the EDHS was nationwide done on a larger sample. In addition, the EDHS was conducted on culturally different population, which may inhibit child feeding practices. While the current study was conducted on almost culturally homogenous population with similar feeding practices.

However, our finding is lower than the national nutrition survey report of Afghanistan (27.6%) [16], Pakistan (56.4%) [25], Vietnam (71.6%) [4], Zimbabwe (28%) [26], and other similar studies in Ghana (34.8%) [27]. The difference could be the low educational level of mothers compared to those of Afghanistan, Pakistan, Vietnam and Ghana. Uneducated mothers may not easily understand the consequences of undiversified diet and the nutritional requirements of infant and young children. According to previous studies, maternal education is one of the key determinant factors for practicing the minimum dietary diversity [14, 15, 19].

The proportion of children who the received minimum meal frequency was 72.2% (95% CI: 69.3, 75%). The finding is also higher compared with the demographic and health survey (DHS) reports of Afghanistan 52.1% [16], Pakistan 56.4% [25], Zimbabwe 59% [26], Indonesia 58.2% [28], Malawi 53.5% [29], and Ghana 57.3% [27]. This difference might be due to the fact that most of the surveys in Asian countries used DHS data, secondary data analysis which contain large populations with ethnic, religious, cultural, belief, and traditional variations. Whereas, due to better food production and high purchasing power is a responsible factor for higher meal frequency practice than Zimbabwe, Malawi, and Ghana [30]. But the practice is lower compared to that Vietnam (85.6%) [4], and the finding of similar studies in Kolkata (78%) [31], Zimbabwe (91%) [26],and Kenya (85.3%) [22]. The difference could be due to lack awareness of mothers about how many times they should give solid, semisolid, and soft food to their children and lack of food stability. In addition, caregiver’s level of encouragement during feeding may contribute to low prevalence [30].

Mothers involved in the monthly growth monitoring follow-ups were more likely to provide the recommended dietary diversity and meal frequency compared to their counterparts. A similar finding was reported from Southern Ethiopia [18]. This could be due to growth monitoring and promotions supported by individual counseling and community conversations which are likely to enhance the understanding of mothers about how to prepare and feed their children with diversified foods. In addition, the monthly growth monitoring program is supported by practical demonstrations on complementary food preparation.

Mothers with satisfactory media exposure were about 2.8 times more likely to practice adequate dietary diversity compared to mothers with unsatisfactory media exposure. This finding is in line with another finding in Ethiopia [14, 15]. Because media promotion enhances timely, adequate, safe,and proper feeding practices. Moreover, the Ethiopian Ministry of Health and its partners distribute radio and family health cards to the health development army, which is an important tool for improving dietary diversity practices of the community.

Postnatal care visit was significantly associated with infant and young child feeding practices. Accordingly, mothers who had postnatal care visits were more likely to provide the recommended dietary diversity and meal frequency compared to their counterparts. This finding is supported by the finding from Sri Lanka [19]. This could be due to the fact that nutritional advice and counseling by health workers might not only educate mothers but also avoids traditional beliefs that might inhibit child feeding practices.

In this study, mothers in the 25–34 years age group were about 2 times more likely to provide diversfied foods compared to mothers in the 15–24 years age group. This finding is supported by the 2011 EDHS report [14]. The possible explanation is that mothers in this age group had a better educational status (69.2%), college diploma and above, and a high proportion,(59.4%) and (53.4%) had satisfactory media exposure and PNC service utilization, respectively.

Likewise, the likelihood of receiving the minimum dietary diversity among children aged 18–23 months was 2.3 times more compared to children aged 6-11 months. This finding is in line with that of EDHS 2011 [14], Dangila [15] and Mekelle [23]. This could be due to the fact that as the age of children increases, the probability of receiving diversified foods increases and the misconceptions of mothers that younger infants and children could not be able to digest food like meat and egg decreases [28]. On tip of that, advances in the age of the children may encourage mothers to initiate complementary feeding as observed in this investigation.

Wealth status of the family was one of the determinant factors for meal frequency. Accordingly, children belonging to middle and rich wealth status families were about 2 times more likely to receive the minimum meal frequency as compared to children belonging to poor wealth status families. This result is similar to that of study conducted in Dangila town, Ethiopia [15] and south Asiatic countries like India, Sir lanka, and Nepal [19]. This may due to the fact that middle and rich wealth status families are more likely to be food secure and have the ability to purchase different consumables goods for their families.

Mothers lived in the urban area were more likely to provide the recommended minimum meal frequency as compared to mothers who lived in the rural area. The possible explanation is that mothers who live in the urban area have good awareness on feeding practices of infants and young children, while urban mothers have access to media which promote the importance of complementary feeding practices as compare to rural mothers. In addition to these access to markets for consumption goods, food and to places of income-generating employment is essential in urban area.

Mothers who obtained information from health professionals were more likely to provide the recommended meal frequency compared to their counterparts. This finding is similar to that of a study conducted in India [32]. This is because health professionals can transmit the appropriate messages on child feeding practices during the critical contact time.

Children in the age group of 12–17 months and 18–23 months were about 2 and 8 times more likely to receive the recommended meal frequency as compared to children in the age group of 6–11 months. This finding is supported by that of another study conducted in Ethiopia [14]. This is probably because infants 6–8 months old are mostly breastfed, so the need for a frequent feeding of extra solid food is not perceived as important or a priority by mothers and caretakers for feeding infants of this age. In addition, older children have the chance of eating family diet, which increases feeding frequency.

The study attempted to show child feeding practices in a well-defined population representing rural northwest Ethiopia. However, some of the limitations of this study should be taken into consideration. First, the study did not consider the quantity of food consumed by the children and a single 24-h recall did not indicate the usual dietary habit of the children. Second, even though adequate training was given to field assistants (data collectors and supervisors) and mothers were clearly informed about the objectives of the study, there might still be a social desirability and recal bias in reporting the type of food given to children.

## Conclusion

This study revealed that only a few children received dietary diversity and meal frequency as measured by the WHO indicators. Postnatal care visits, age of child, and growth monitoring participation of mothers affect both minimum dietary diversity and meal frequency practices. Lack of media exposure and younger age of mothers affect minimum dietary diversity, while rich wealth status of household, residence, and information from health professional were associated with meal frequency. Hence, encouraging all mothers to participate in monthly growth monitoring, intensive media advertising and strengthening counseling of mothers attending PNC are proposed for achieving the recommended infant and young child infant and young child feeding practices.

## Abbreviations

ANC: Antenatal Care
AOR: Adjusted Odd Ratio
CF: Complementary Feeding
CI: Confidence Interval
COR: Crude Odd Ratio
EDHS: Ethiopian Demographic and Health Survey
FMOH: Federal Ministry of Health
IYCF: Infant and Young Child Feeding
MDD: Minimum Dietary Diversity
MMF: Minimum Meal Frequency
PNC: Postnatal Care
UNICEF: United Nations Children Fund
USAID: United State Agency for International Development
WABA: World Alliance for Breastfeeding Action
WHO: World Health Organization
